# Analyses of the Mineral Waters near Buffalo, and Remarks

**Published:** 1846-02-01

**Authors:** E. R. Long

**Affiliations:** United States Army


### Analyses of the Mineral Waters near Buffalo, and Remarks.

Communicated by Lieut. E. R. LONG, M. D., United States Army.

DETROIT, Jan. 2, 1846.

DEAR SIR:—Having, as you are aware, devoted some little attention to the analysis of the mineral waters in the vicinity of Buffalo, I give below the results of my investigations. Imperfect as they are, if they serve to direct more general attention among the good people of your city to the medicinal virtues of these springs, I will esteem myself amply remunerated for my labor. And having, as I conceive, derived much personal benefit from their use, I feel in a measure bound to proclaim, publicly, their merits. Many years a victim of dyspepsia, and, as a matter of course, passing in that period through the whole materia medica, I can speak from experience; and, if I have not greatly deceived myself, I have been as much benefited by the water of the Indian Reservation Spring, as by any other remedy I have tried. As to the modus operandi of this water, or, as to its most efficient medicinal ingredient, there may exist some difference of opinion. But, for the reasons given in the sequel, there is but little doubt that its chief virtue may be fairly ascribed to its Sulphureted Hydrogen. Although I analyzed this water myself, yet from the imperfections of my chemical apparatus, I did not attempt a quantative analysis; I will, therefore, give that of Dr. Chilton, of New York, which I found to correspond with my own in all that relates to the number and character of its several ingredients. I also insert the analyses of some other springs, with a view of determining, by comparison, their relative merits as medicinal agents:

*Spring on the Indian Reservation*—by Dr. Chilton.*

| | | | |
|---|---|---|---|
| Chloride of Sodium, | 86.40 | grains in 1 gallon. | |
| " of Calcium, | 20.00 | " | " |
| " of Magnesium, | 16.80 | " | " |
| Carbonate of Lime, | 10.88 | " | " |
| " of Soda, | 1.28 | " | " |
| Sulph. Magnesia, | 6.56 | " | " |
| " Lime, | 3.36 | " | " |
| Vegetable matter, | .41 | " | " |
| Total, | 154.01 | solid contents. | |

* This Spring is located about four miles from this City.

| | | | |
|---|---|---|---|
| Gases—Sulphureted Hydrogen. | 24.06 | cubic inches. | |
| Carbonic Acid, | 6.00 | " | " |
| Total, | 30.06 | | |

*Avon, Lower Spring—by Dr. Chilton.*

| | | | |
|---|---|---|---|
| Carbonate of Lime, | 29.23 | grains in 1 gallon. | |
| Chloride of Calcium, | 8.41 | " | " |
| Sulph. of Lime, | 57.44 | " | " |
| " of Magnesia, | 49.61 | " | " |
| " of Soda, | 13.73 | " | " |
| Total, | 158.52 | solid contents. | |
| Gases—Sulphureted Hydrogen, | 10.02 | cub. inch. in 1 gal. | |
| Nitrogen, | 5.42 | " | " |
| Carbonic Acid, | 3.92 | " | " |
| Oxygen, | 56 | " | " |
| Total, | 20.02 | | |

*White Sulphur Springs, Va.—by Prof. Rogers.*

| | | | |
|---|---|---|---|
| Sulph. Magnesia, | 22.32 | grains in 1 gallon. | |
| " Lime, | 30.80 | " | " |
| Carb. " | 4.04 | " | " |
| Mur. " | .80 | " | " |
| Chloride of Sodium, | .40 | " | " |
| Total, | 60.80 | solid contents. | |
| Gases—Sulphureted Hydrogen, | 2.05 | cub. inch. in 1 gal. | |
| Carbonic Acid, | 2.00 | " | " |
| Oxygen, | 1.44 | " | " |
| Nitrogen, | 3.55 | " | " |
| Total, | 9.49 | | |

*Aix La Chapelle—a Sulphur Spring.*

| | | |
|---|---|---|
| Solid constituents in 1 gallon, | 86.00 | grains. |
| Gases—Sulphureted Hydrogen in 1 gallon, | 22.00 | cubic inches. |

*Seltzer—a Carbonated Spring.*

| | | | |
|---|---|---|---|
| Carbonate of Soda, | 16.00 | grains in 1 gallon. | |
| " of Magnesia, | 20.00 | " | " |
| " of Lime, | 12.00 | " | " |
| Chloride of Sodium, | 68.00 | " | " |
| Total, | 116.00 | | |

Before instituting a comparison between these several waters, it may be well to ascertain, as near as may be, the therapeutical principle upon which their efficacy depends. It appears that they abound in neutral salts, and that the most valuable of them are richly impregnated with Sulphureted Hydrogen, or Carbonic Acid gas. It has also been pretty well determined by observation, that artificial mineral waters holding in solution the same salts, in the same proportions, impregnated with the gases, do not possess the same virtues as those from the natural fountains. And it has also been observed, that these latter, when kept some time in open vessels, before use, are not so efficaceous as when taken fresh from the Springs—in fact that they have lost their specific virtue.

And it is a well established fact, that when the system has been long exposed to the action of cathartic, mineral waters, it does not become so reduced in flesh and strength, as by the use of the same salts administered in the ordinary way—a fact fairly explicable by the tonic and invigorating powers of the gases found in the former.

From these facts it is obvious, that the gaseous ingredients of mineral waters confer upon them their most valuable properties. Hence that spring, *cœteris paribus*, that contains the greatest proportion of such ingredients, must be esteemed the more valuable.

On referring to the foregoing analyses, we see that the Indian Res. Spring contains a greater proportion of solid ingredients than the White Sulph. of Va., and almost as much as the Lower Avon Spring, while it contains more than double the quantity of Sulph. Hydrogen, found in either;—hence, as it is probable that the peculiar virtues of this class of mineral waters—the sulphurous—to which these belong, are derived from this gas, it is fair to conclude *a priori*, that it is the best of the three; and I doubt not future observation and experience will fully confirm this conclusion.

But it has been found that the water of this spring does not act as freely upon the bowels as some other mineral waters containing a smaller proportion of purgative salts—which renders it necessary, in some cases, to administer some other aperient in conjunction with the water. It, however, possesses powerful diuretic properties, and will prove very efficient in diseases of the bladder and kidneys, in dropsy, and other complaints requiring a remedy of this character.

I also analysed the water of two springs on the farm of Wm. Armes, near Black Rock, and one near Williamsville. They all belong to the class of carbonated waters, being highly impregnated with carbonic acid, and holding in solution an excess of the carb of lime, and carb. of magnesia, which, as soon as the gas escapes into the atmosphere, is deposited in white powder near the springs, or in porous masses of a greyish, or brownish color on sprigs of moss, or branches of trees in, or near the springs. These waters hold in solution, besides the carbonates of lime and magnesia, sulphates of the same bases—no traces of soda, potassa, iron, iodine, or bromine could be discovered by the usual tests. They contain about 128 grs. of salts to the gallon, which is a higher proportion than is found in either the Seltzer Spa, or Pyrmont waters, belonging to the same class; and as they doubtless possess as much carb. acid, they are in all respects equal to the latter in curative properties.

In conclusion, I will add that the sulphurous waters, like the Indian Reservation Spring, are indicated in diseases of the digestive organs; also in scrofulous and rheumatic affections; but more especially in cutaneous affections, from the specific action of the sulph. hydrogen on the skin.

The carbonated waters, similar to those of the Black Rock and Williamsville Springs, have tonic and diuretic properties, and are serviceable in dyspepsia, gout, and hypochondriasis; also in the debilitated and emaciated condition of the system consequent upon attacks of acute disease, or long sieges of bilious and other fevers.

I will state, for the benefit of those who are desirous of using these waters, and cannot conveniently do so at the Springs, that they may, with proper care, preserve the water in barrels so as to retain most of its gases. This is done by drawing the water off by a stop-cock at the lower part of the barrel, without a vent at the top. This is rather a tedious process, but if a vent be made at the top of the barrel, the water will soon loose most of its virtue.

Yours truly, E. R. LONG.

